# An ultrasensitive microfluidic approach reveals correlations between the physico-chemical and biological activity of experimental peptide antibiotics

**DOI:** 10.1038/s41598-022-07973-z

**Published:** 2022-03-07

**Authors:** Jehangir Cama, Kareem Al Nahas, Marcus Fletcher, Katharine Hammond, Maxim G. Ryadnov, Ulrich F. Keyser, Stefano Pagliara

**Affiliations:** 1 Living Systems Institute, University of Exeter, Stocker Road, Exeter, EX4 4QD UK; 2 College of Engineering, Mathematics and Physical Sciences, University of Exeter, North Park Road, Exeter, EX4 4QF UK; 3 Cavendish Laboratory, Department of Physics, University of Cambridge, JJ Thomson Avenue, Cambridge, CB3 0HE UK; 4 National Physical Laboratory, Hampton Road, Teddington, Middlesex, TW11 0LW UK; 5 London Centre for Nanotechnology, University College London, London, WC1H 0AH UK; 6 Department of Physics, King’s College London, Strand Lane, London, WC2R 2LS UK; 7 College of Life and Environmental Sciences, University of Exeter, Stocker Road, Exeter, EX4 4QD UK

**Keywords:** Biochemistry, Biological techniques, Biophysics, Drug discovery, Microbiology

## Abstract

Antimicrobial resistance challenges the ability of modern medicine to contain infections. Given the dire need for new antimicrobials, polypeptide antibiotics hold particular promise. These agents hit multiple targets in bacteria starting with their most exposed regions—their membranes. However, suitable approaches to quantify the efficacy of polypeptide antibiotics at the membrane and cellular level have been lacking. Here, we employ two complementary microfluidic platforms to probe the structure–activity relationships of two experimental series of polypeptide antibiotics. We reveal strong correlations between each peptide’s physicochemical activity at the membrane level and biological activity at the cellular level. We achieve this knowledge by assaying the membranolytic activities of the compounds on hundreds of individual giant lipid vesicles, and by quantifying phenotypic responses within clonal bacterial populations with single-cell resolution. Our strategy proved capable of detecting differential responses for peptides with single amino acid substitutions between them, and can accelerate the rational design and development of peptide antimicrobials.

## Introduction

Antimicrobial resistance is the “silent” pandemic that threatens to undermine modern medicine. Without intervention, it is predicted to cause 10 million deaths per year by 2050^1^, with losses to the global economy of up to $3.4 trillion p.a. within the next decade^2^. A combination of scientific and economic challenges^3–5^ has led to the drying up of the existing antimicrobial pipeline. Given the vital role that antimicrobials play in modern healthcare, it is crucial to develop and maintain new pipelines of effective antimicrobials, particularly with drugs that can circumvent currently prevalent resistance mechanisms.

Antimicrobial peptides (AMPs) have attracted significant interest as alternatives to traditional small molecule antibiotics^6–9^. In contrast to conventional antibiotics, there is no widespread resistance to these evolutionarily conserved molecules. AMPs are killing factors of innate immune systems of all life forms, which modulate host immune responses to clear infections^10^. Bacteria that are resistant to widely employed antibiotics have been shown to have a high frequency of collateral sensitivity to AMPs^11^. AMPs also show anti-biofilm activity, besides having anti-inflammatory and wound-healing properties^12^. In particular, cationic peptides have been designed to mimic the antimicrobial activity of α-helical host defence peptides that form an important part of the antimicrobial defences of multicellular organisms. These cationic peptides selectively target and attack the anionic membranes of bacterial pathogens^13,14^. Encouragingly, a recent update from the CARB-X antibiotic accelerator reports that direct-acting peptides account for approximately 11% of their portfolio, showing renewed industry interest in these compounds to overcome multidrug resistant bacterial strains^15^.

However, the clinical translation of peptide antimicrobials has suffered from a lack of suitable assays to characterize their efficacy^16^. Standard bulk techniques such as Minimum Inhibitory Concentration (MIC) assays fail to uncover heterogeneities in drug activity within a population of genetically identical cells. Such cell-to-cell variability is a particular challenge when assessing the antimicrobial efficacy of peptides due to the inoculum effect^17^. Charged peptides can remain bound to cellular components after cell death and lysis, and hence affect the “free” peptide concentration^18^ observed by other cells in the vicinity, leading to heterogeneities in the response to the drug dose; this leads to some cells surviving the treatment in the absence of genetic resistance. We note that the inoculum effect has been observed with certain small molecule antibiotics as well, such as the cephalosporin class of beta-lactam antibiotics^19^; approaches that can circumvent this effect thus have utility beyond the study of peptide based drugs. Cationic peptides also adsorb to surfaces of glass and plastic, leading to a loss of free peptides from solutions^20^; this complicates experimental handling and may lead to inaccuracies in data interpretation in standard assays. A recent study by the Stella group confirmed a strong inoculum effect on the MICs of a range of antimicrobial peptides, which led the authors to question the utility of standard cellular screening assays for the field^21^. Other researchers have also identified a need for “new and standardized testing structures” to facilitate the translation of peptide antimicrobials from the bench to the clinic^16^. In their comprehensive review on the subject, Mercer et al. list a range of factors that can additionally influence the activity of AMPs, such as media composition, diluents, additives, the pH and ionic strength of test solutions, the presence of metal ions, amongst others^22^; these all need careful consideration during the development of standardized testing protocols. Further, given the fact that peptides sometimes have multiple targets, and in particular often attack bacterial membranes, characterization strategies should also include tests for membrane activity, in addition to cellular assays.

We recently reported the development of a novel set of ultrashort (≤ 11 amino acids) peptides, based on a minimal amphipathic helix with *bi*nary *en*coding (bien) by arginine and leucine, that showed antibacterial efficacy^23^. We refer to these compounds as “peptide” or “polypeptide” antibiotics, based on historical terminology used by Hancock^24^, also considering that these are synthetic peptides rather than natural AMPs^25^. Notably, a single side-chain mutation in the hydrophobic face of the helix significantly changed the nature of peptide-lipid interactions leading to differential mechanistic and biological responses. An alanine at the mutation position (“bienA”) facilitated a deep insertion of the helix in lipid membranes, and the formation of circular pores in (anionic) supported lipid bilayers (SLBs). However, a lysine at the same position (“bienK”) led to a shallower insertion of the helix and the formation of fractal membrane ruptures in the SLBs (peptide sequences are provided in Table 1)^23^. To probe the connection between the biological (cellular) and physico-chemical (membranolytic) properties of these new peptides, here we study the interaction of the bienA and bienK peptides with hundreds of individual *Escherichia coli* cells using the microfluidic “mother-machine” device^26^, and in parallel investigate their interactions with model membranes in a bespoke assay on hundreds of giant unilamellar vesicles (GUVs)^27^. The use of these high-resolution platforms enabled us to reveal phenotypic variants that survived each of these new compounds and revealed important insights about their activity both at the membrane and cellular level.

**Table 1:** List of peptides studied with their corresponding amino acid sequences and mass-to-charge (m/z) ratios as calculated and found via MALDI-ToF mass spectrometry.

Peptide name	Peptide sequence (mutation position highlighted in bold)	m/z (calculated)	m/z (found)
Bien A9	RLLRL**A**LRL	1122.4	1125.0
Bien A10	RLLRL**A**LRLL	1235.6	1238.4
Bien A11	RLLRL**A**LRLLR	1391.8	1395.6
Bien K9	RLLRL**K**LRL	1179.5	1182.2
Bien K10	RLLRL**K**LRLL	1292.7	1295.6
Bien K11	RLLRL**K**LRLLR	1448.9	1451.2

## Results and discussions

**Figure 1:**
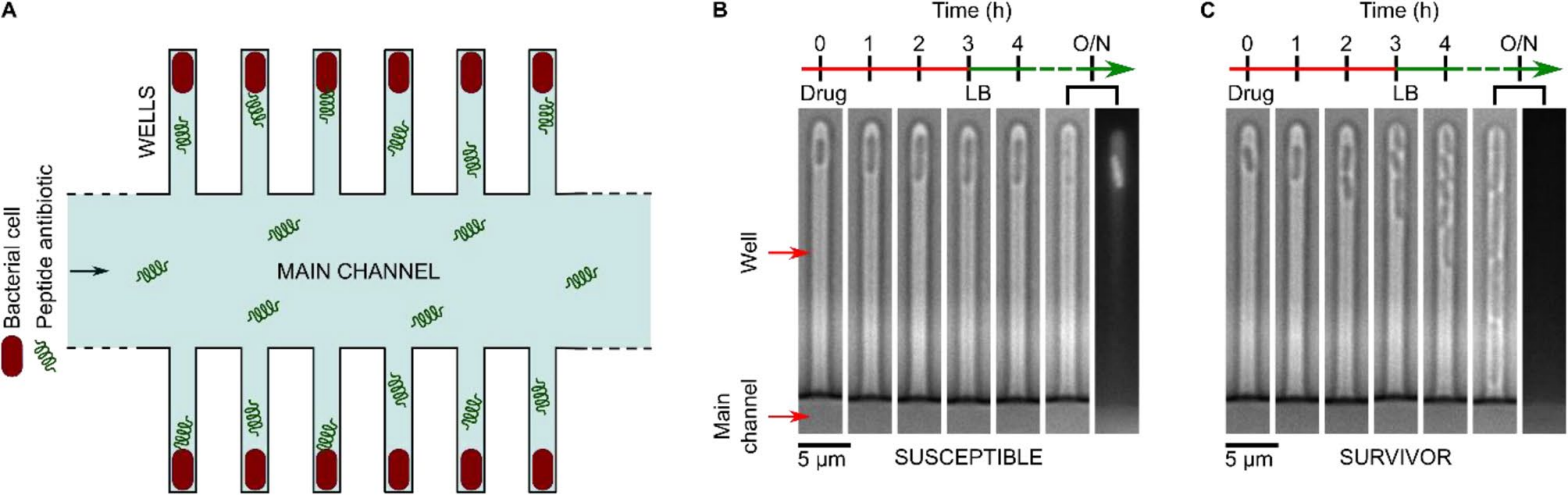
Probing the biological activity of experimental polypeptide antibiotics with single-cell resolution. The mother-machine microfluidic device, whose schematic is shown in (**A**), consists of a “Main channel” for seeding the connected side channels or “wells” of the device with cells. Drugs, nutrients, and viability stains are then dosed to the trapped cells via the main channel. In the microscopy images tracking individual wells in (**B**) and (**C**) above, we observe examples of individual susceptible (**B**) and survivor (**C**) cells in response to peptide treatment in the mother-machine microfluidic device. The two examples above are taken from the same experiment and show the contrasting responses of two clonal *E. coli* cells to the peptide bienK11 (10 µM). The cell shown in (**B**) was susceptible to the treatment, halting growth during the 3 h of drug dosage and disintegrating thereafter when the drug was replaced with fresh Lysogeny Broth (LB) and incubated overnight (O/N). The disintegration is clearly visible when comparing the 4 h and O/N time-points—in the final fluorescence image panel, taken after O/N LB treatment, we see the debris of the cell stained with the dead stain propidium iodide (PI). In contrast, the cell shown in (**C**) resisted the treatment, growing and dividing even during peptide delivery, with no PI staining in the daughter cells at the end of the experiment.

The “mother-machine” system (Fig. 1A) enables the physical confinement of individual bacteria in well-controlled microenvironments, where they can be sequentially exposed to nutrients, drugs or viability indicators and the responses studied using time-lapse microscopy^28–30^. This technology has been used extensively to study bacterial-drug interactions, using a range of conventional small-molecule antibiotics such as ampicillin, gentamicin, ciprofloxacin (amongst others)^28,30–33^, phages^34^ as well as novel protein pseudocapsid based antibacterials^35^. Briefly *E. coli* BW25113 cells were cultured overnight in Lysogeny Broth (LB) at 37 °C to stationary phase and injected into the mother-machine microfluidic device (see “Experimental section” and^28,29,31,32^). We waited for the cells to populate the “wells” of the chip (Fig. 1), following which the trapped cells were challenged with a continuous flow of peptide (10 μM) for 3 h, with images taken hourly. After 3 h of treatment, the cells were provided with fresh LB to study survivors. Post overnight (O/N) LB treatment, dead staining was performed with propidium iodide (PI). Dead cells either lysed or took up PI and appeared bright under fluorescence illumination (Fig. 1B). The survivors could be further categorised into those that were dividing (Fig. 1C), and those that did not stain with the dye but did not divide either (Fig. S1, Table S1) as previously reported^30–32^. As a caveat, we note that there is some debate in the literature about these non-dividing cells that do not stain with PI; some groups claim that they are simply dead cells^36^, while others have found them to be viable^30,31,37,38^. However, the cells that were dividing at the end of the experiment were unambiguously alive. Therefore, we chose to use only these dividing cells in our quantitative analysis discussed below.

**Figure 2:**
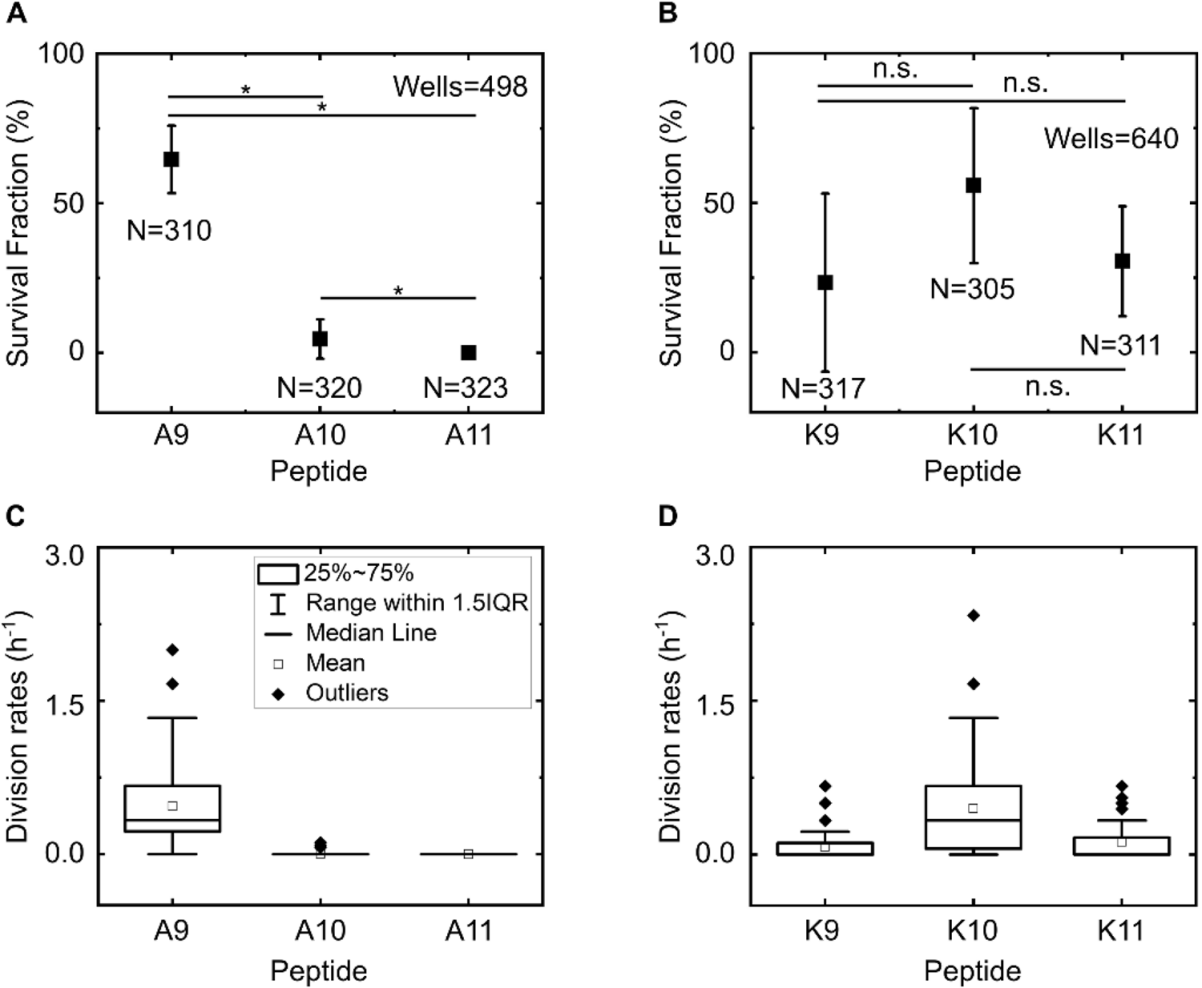
Single amino acid substitutions dramatically alter the efficacy of bien peptides at the single-cell level. From both the fraction of wells hosting dividing cells at the end of the experiment, (defined as the *survival fraction*, **A**,**B**) and the cell division rates during peptide treatment measured at the level of individual wells (normalized to the number of cells initially hosted by the well, **C**,**D**), we observed that the peptides bienA11 and bienA10 were the most potent of the set, with complete inhibition of division for bienA11 and a similar result for bienA10. In the bienA series, reaching a critical peptide length led to a marked increase in peptide activity, but this was not the case for the bienK series. BienA9 and bienK10 showed similar levels of activity, but were the least potent with over 50% of the wells showing cells that divided despite the treatment. BienK9 and bienK11 showed more antimicrobial activity on average, but with considerable heterogeneity in the responses. Note, we typically only analysed wells that hosted between 1 and 3 cells each at the start of the experiment to avoid difficulties with tracking if the cells started growing, dividing, and exiting the channel during the drug treatment (this was not a concern for the bienA11 and bienA10 experiments since no/minimal cell division was observed in response to these peptides). We did not detect any potential bias on the survival fraction due to the initial number of cells in the well (SI Fig. S2). Results are shown for two independent experiments (i.e., two independent culture isolates in independent technical repeats) for each treatment. We analysed at least 150 individual cells for each peptide experiment, so we studied over 300 individual cells per peptide across the 2 biological repeats (as mentioned inset in **A** and **B**). All peptides were dosed at 10 µM concentrations. Plots in (**A**) and (**B**) report mean ± std. dev. For comparison, in our no-peptide control experiments, the survival fraction was 91 ± 11% (162 cells tracked across 2 biological repeats). Statistical significance tested at the 0.05 level using a 2-sample t-test with Welch’s correction (for p-values see SI Table S2).

We defined survival fraction as the fraction of wells hosting dividing cells after the 3 h peptide treatment and subsequent incubation in LB overnight (typically 17–18 h of LB incubation in total, see “Experimental section”); as noted above, these dividing cells were unambiguously alive. This is equivalent to the survival fraction measured via colony forming unit (CFU) bulk assays and we therefore used this as a quantitative metric for the inhibitory efficacy of the peptides^30^. The bienA11 and bienA10 (hemolytic^23^) peptides were clearly the most potent in our assay, with a survival fraction of less than 5% (Fig. 2A). In contrast, the bienA9 peptide was less effective with a survival fraction of 65 ± 11% (mean ± std. dev. over 2 biological repeats, 199 wells, 310 cells). Thus, reaching a critical peptide length leads to increased antibacterial activity with the bienA peptides. However, we did not find a similar dependence between antimicrobial activity and peptide length for the bienK series, where we also observed a high frequency of phenotypic variants surviving peptide treatment. For bienK10 and bienK11, the survival fractions were 56 ± 26% (204 wells, 305 cells) and 31 ± 18% (215 wells, 311 cells) respectively (Fig. 2B), markedly different from the bienA10 and bienA11 results (Fig. 2A). Thus for these bienK peptides, while some individual cells were killed by the peptides, a large number of other clonal relatives survived the exact same conditions, similar to the example shown in Fig. 1. Unlike traditional CFU or MIC assays, the peptides are dosed continuously for 3 h, and hence there should not be any significant differences around peptide exposure for the survivor versus susceptible cells and no inoculum effect (Fig. S2). Our results reveal important phenotypic variants seemingly oblivious to the effects of these polypeptide antibiotics that are able to grow and divide even during treatment, while their clonal relatives perish. We also observed similar trends when quantifying the cellular division rates in response to the peptides (Fig. 2C,D). The bienA11 and bienA10 peptides immediately and uniformly arrested cell division in all experiments, whereas bienA9 did not (Fig. 2C), recapitulating the peptide length dependence observed for the survival fraction. Again, as with the survival fractions, this peptide length dependence was not observed for the cellular division rates with the bienK series (Fig. 2D).

**Figure 3:**
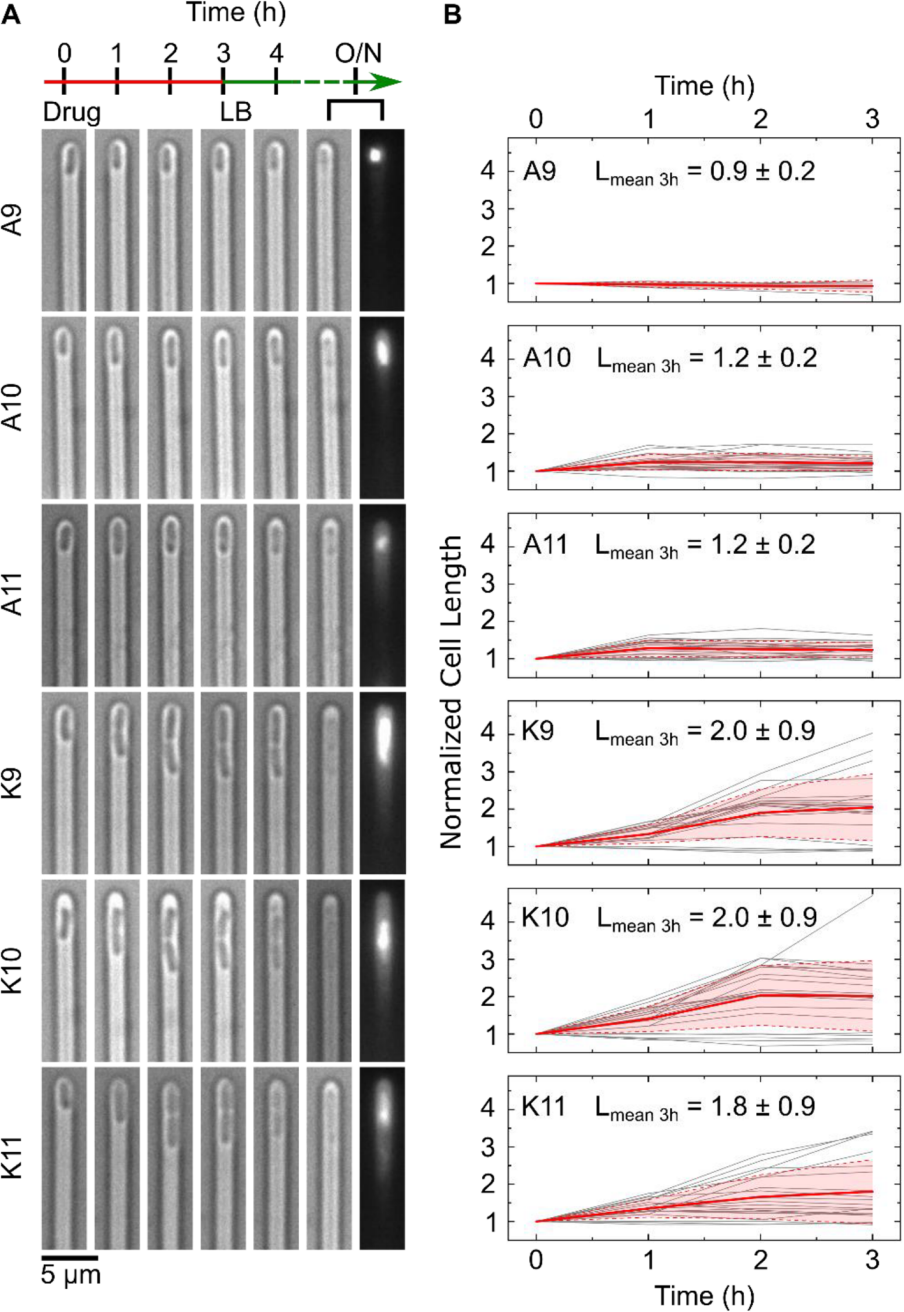
Comparing the single-cell killing phenotypes of our two experimental peptide series. The single-cell microscopy images in (**A**) depict differences in the killing phenotypes between the bienA and bienK peptides. The first image in each set (at *t* = 0) refers to the point at which drug dosage starts, with the next images showing the cell at hourly intervals over 3 h of drug treatment (all peptides were dosed at 10 µM concentrations). The following image shows the cell after 1 h of LB incubation, followed by images showing the debris of the dead cell after overnight (O/N) incubation in LB (bright-field and corresponding PI stain fluorescence image). The bienA series kill the cells rapidly, with the cells showing minimal elongation before growth arrest and death. This is also apparent from the corresponding graphs, shown in (**B**), tracking the lengths of individual cells (lengths are normalized to the initial cell length). Individual cell traces are provided in grey, with the mean (red) and standard deviation (red shaded area) depicted. The cellular length changes (before death) and distributions are relatively homogeneous for the bienA peptides. In stark contrast, the bienK peptides showed much greater heterogeneity in the lengths. Interestingly, for all three bienK peptides, we observed cells dying during septation. However, as mentioned previously and as seen in the plots in (**B**), this was not the only phenotype. On average, with the bienK peptides, cells doubled in length before growth arrest and death, but there was significant spread in the lengths, as shown in the mean lengths (normalized) at the 3 h time-point reported inset in each plot (mean ± std. dev.). Note, for this analysis, we studied a subset of cells that were susceptible to the peptides, and were killed before undergoing any cell division. We chose a subset of 20 cells from 2 independent biological repeats for all the peptides, with the exception of bienA9 (5 cells, 1 experiment) which had far fewer cells showing the necessary characteristics. To reiterate, these subsets of cells were chosen according to the following criteria—(i) the cells were susceptible to the drug, (ii) they died before dividing and (iii) they did not disintegrate immediately after treatment, since we wished to track their length over the entire course of drug treatment (3 h).

Next, we sought an explanation for this phenotypic variability and investigated the mode of killing activity across the clonal population. When challenged with the bienA peptides, the cells showed rapid growth arrest; typically, no growth was seen after the 1 h observation time-point (Fig. 3). In striking contrast, for cells treated with the bienK series, cells often elongated and septated until the point of division, when they appeared to stop growth and die (Fig. 3A). This suggests a killing mechanism associated with the inhibition of processes involved in cell division. As noted in Fig. 3B, cells treated with bienA10 and bienA11 showed a 20% increase in length on average before cell death, whereas with the bienK series cells doubled in length (on average) before death. This is possibly linked to the membrane activity of the bienK peptides, which were found in supported lipid bilayer (SLB) experiments to disrupt the outer leaflet of lipid bilayers^23^; these peptides may be exploiting weaknesses in the integrity of the membrane during the complex division process. However, there was significant variability in the response to each of the bienK peptides, and not all cells showed the same phenotype. For the cells analysed in Fig. 3B, at the 3 h time point, the coefficient of variation (CV) in the lengths was 17%, 16% and 16% for the bienA9, bienA10 and bienA11 experiments respectively. For bienK9, bienK10 and bienK11, the CV values of the lengths were 44%, 47% and 48% respectively, showing the greater heterogeneity in lengths (at point of death) in response to each of the bienK peptides as compared to the bienA compounds. It is worth mentioning that the human antimicrobial peptide LL-37 has also been shown to attack and kill septating *E. coli* cells, where peptide binding was found to be preferential near the septum of the cells^39^; further mechanistic investigations using complementary techniques may help elucidate whether there are similarities between the molecular mechanisms of action of these compounds.

**Figure 4:**
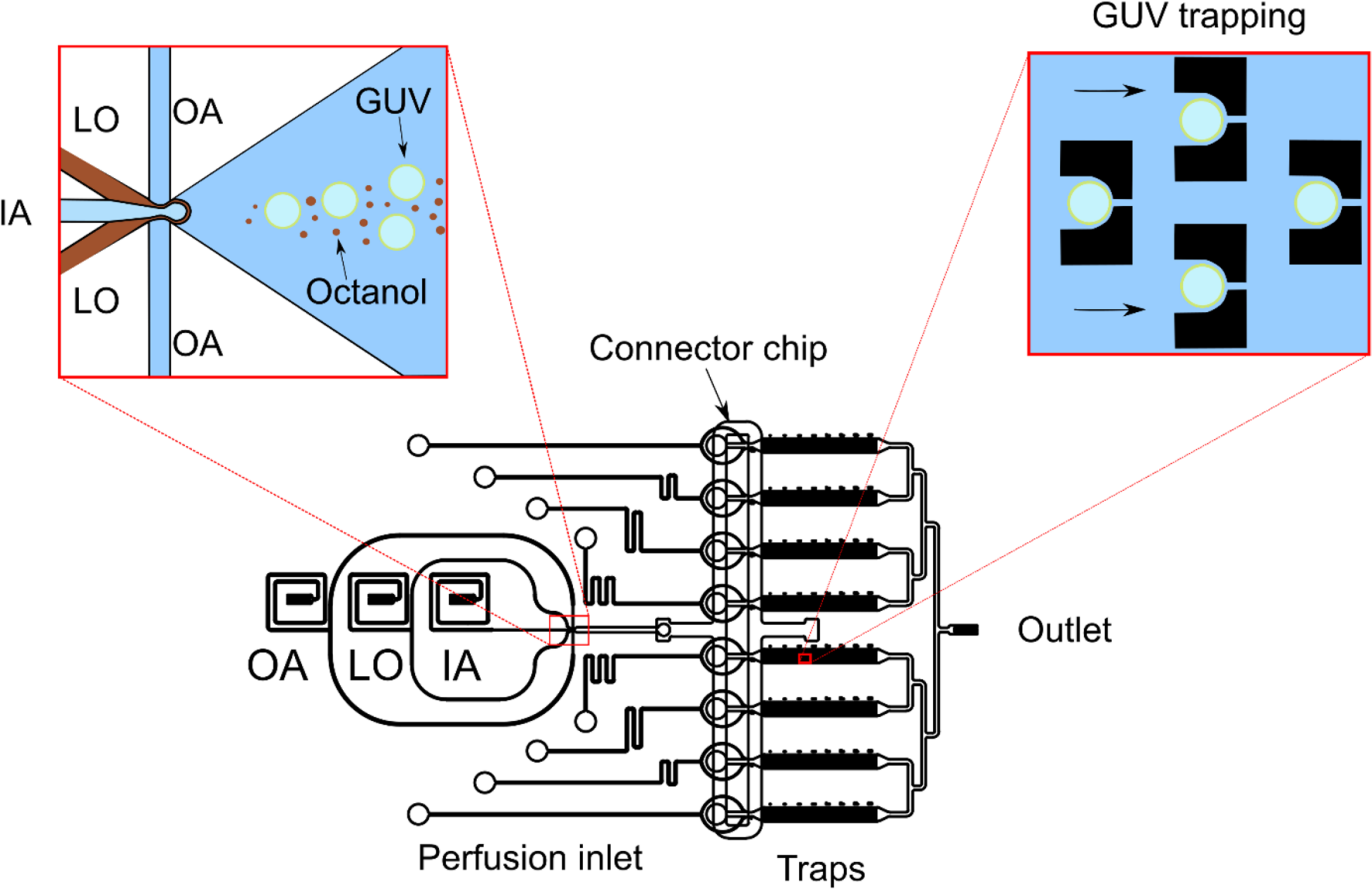
Schematic of the integrated GUV production, trapping and perfusion platform^27^. GUVs are produced using octanol-assisted liposome assembly at a 6-way junction as described elsewhere^40^. Briefly, lipids dissolved in octanol (the “LO” phase) are brought together at the junction with inner aqueous (IA) and outer aqueous (OA) streams, leading to the production of GUVs hosting octanol droplets. The octanol droplets bud off the GUVs downstream. This mixture of GUVs and octanol droplets is distributed into an array of trapping chambers via a connector chip, which additionally facilitates the separation of the GUVs and octanol droplets (for details, please see Al Nahas et al. *Lab Chip* 2019^27^). The trapped vesicles contain the membrane-impermeable fluorescent dye HPTS. Post-trapping, the GUVs are subjected to a continuous flow of peptides, and the membranolytic efficacy of the peptides is determined by studying the leakage of the fluorescent dye from the interior of the GUVs via video fluorescence microscopy^27^.

Given this variability in the cellular responses to treatment, we hypothesized that cell-to-cell differences at the membrane level could underlie the observed heterogeneity in the bacterial responses to these novel peptides. We therefore used anionic giant unilamellar vesicles (GUVs) as highly controlled model membranes to study the membranolytic activity of the bienA and bienK peptides^27^. The vesicles were produced in a microfluidic chip using octanol-assisted liposome assembly (OLA)^40^ and made with a 3:1 mixture of 1,2-dioleoyl-sn-glycero-3-phosphocholine (DOPC) and 1,2-dioleoyl-sn-glycero-3- phospho-rac-(1-glycerol) sodium salt (DOPG) to mimic the anionic charge of bacterial membranes^27^ (Fig. 4). We note that *E. coli* membranes are estimated to be ~ 20% PG, and that lipids of the inner membrane in particular contain a high proportion (over 95%) of phospholipids^41^, similar to our phospholipid-based GUV models. The more complex *E. coli* outer membrane includes both phospholipids and glycolipids^41^. Membrane models have proved extremely useful for studying the interactions of AMPs with membranes, showing excellent correlation with the in vivo activity of these drugs^42–44^. In particular, in a direct comparison, AMP induced membrane permeabilisation was similar across GUVs and *E. coli* inner (cytoplasmic) membranes, albeit with some interesting dissimilarities in a few specific details^43^. In our assay, the GUVs were formed encapsulating a membrane impermeable dye, HPTS (8-hydroxypyrene-1,3,6-trisulfonic acid) and immobilized using an array of physical traps; the peptide solution was then flowed over them using a continuous flow system^27^. GUVs whose membranes are disrupted by the peptides lose their fluorescence and hence the system enables us to quantify the membranolytic efficacy of the peptides on hundreds to thousands of GUVs with single-vesicle resolution.

**Figure 5:**
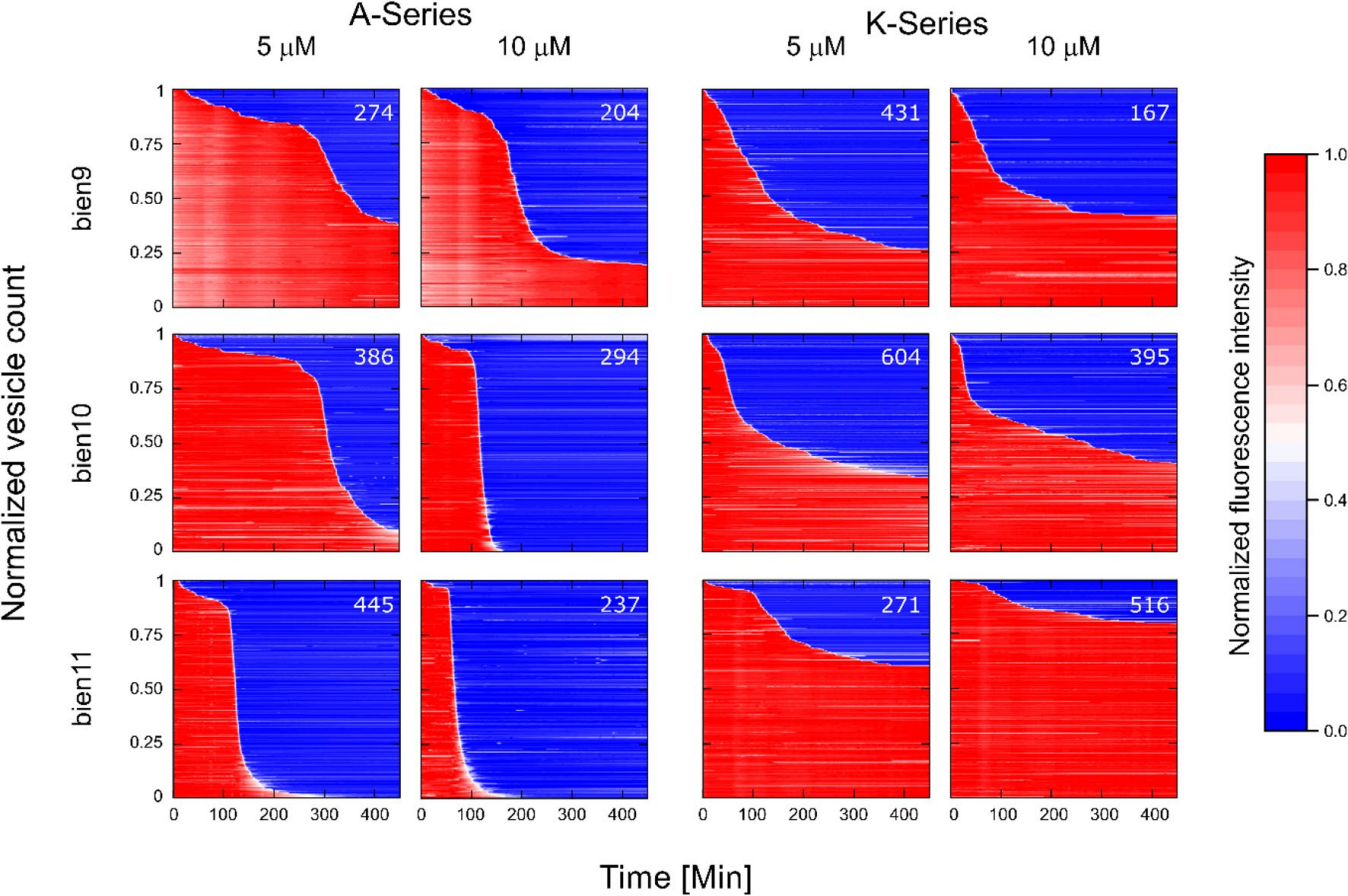
A summary of the membranolytic activity of the bien peptides on populations of bacterial membrane-mimicking lipid vesicles. Dataset 1 of 2 (Dataset 2 is provided in the SI as Fig. S3). Trapped vesicles were continuously treated with the peptides at 5 μM and 10 μM concentrations and their morphology was observed overnight. Each horizontal line depicts the locally normalized intensity of the fluorescent dye HPTS encapsulated in a single trapped vesicle (with global background subtraction) over time. The vesicle's membrane is considered intact at high fluorescence intensity (red) and compromised at low fluorescent signal (blue). The intensity traces were ordered by the critical viability time point, which is defined as the point when the fluorescence intensity of a vesicle decreases below 50% of its initial intensity. The total number of analysed vesicles is reported in white in the top right corner of every plot. The results show that the bienA series of peptides is membranolytic, with potency increasing as one progresses from bienA9 to bienA11 and with an increase in the respective drug concentrations. However, the bienK series is not obviously membranolytic—there appears to be a weakening of the membranes in relation to controls, but there is no obvious concentration dependence, and the trend appears to be different compared to the bienA series, with bienK11 being the least potent in this dataset.

**Figure 6:**
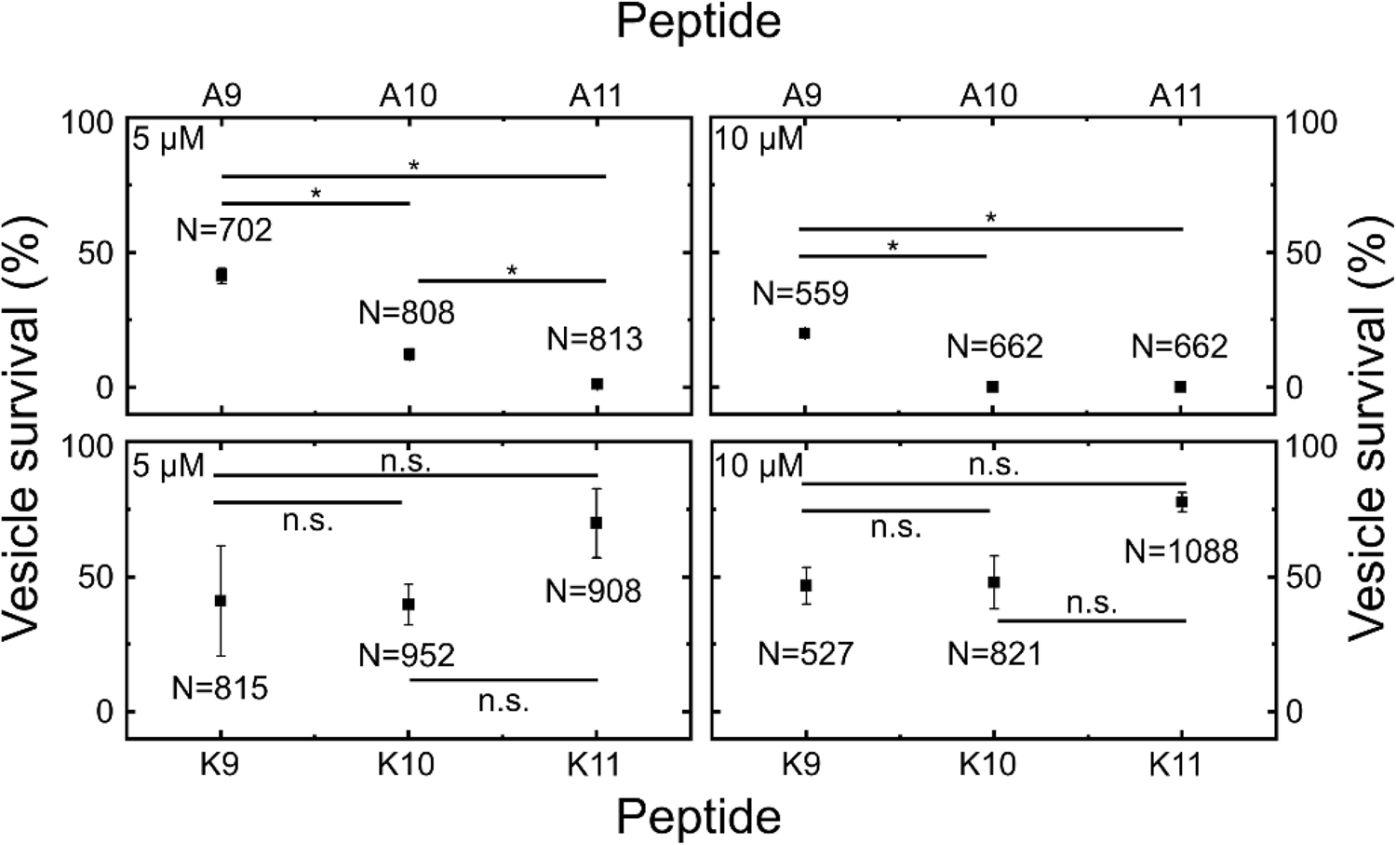
Vesicle viability levels after 7 h of peptide administration*.* Data from two independent repeats per peptide, with total numbers of vesicles analysed mentioned inset (N). Data points represent mean survival percentages, and error bars report the standard deviations. Vesicles were continuously treated with the peptides at 5 μM or 10 μM concentrations in individual chambers each containing hundreds of physical traps. The vesicles in the control chambers were perfused with buffer. The majority of the population was stable under the flow of the control solution, with 86.8 ± 2.2% of the vesicles surviving (mean ± std. dev., N = 633, 2 repeats). BienA11 was the most membranolytic peptide, followed by bienA10. At 5 μM, peptide activity increased with increasing length in the bienA series. At 10 μM, both bienA11 and bienA10 caused complete lysis of the vesicle populations. These trends are in agreement with the single-cell results reported in Fig. 2. However, for the bienK series, none of the peptides led to complete lysis of the population of vesicles, even at 10 μM. Further, the activity of each of the bienK peptides was similar at both 5 and 10 µM concentrations, which indicates a different mode of action as compared to the bienA peptides. We hypothesise that the underlying antimicrobial mechanisms for the bienK series involve membrane weakening, but potentially other intracellular mechanisms as well, based on the activity observed in cells. This requires further investigation. Please note, the single-GUV level data underlying this figure is provided in Figs. 5 and S3. Statistical significance tested at the 0.05 level using a 2-sample t-test with Welch’s correction (p-values in SI Tables S3 and S4). Further, a positive control was performed using the detergent Triton X to validate the GUV platform’s ability to study membranolytic compounds; this data is presented in SI Fig. S4.

We performed experiments at both 5 and 10 μM peptide concentrations. Similarly to the single-cell studies, the single-GUV data showed striking differences between the bienA and bienK peptides (Figs. 5, 6 and S3). In the bienA series, we observed a strong dependence of membranolytic efficacy on peptide length at the 5 μM concentration, with increasing peptide length increasing the membranolytic efficacy by up to 40-fold. In fact, vesicle survival rates (mean ± std. dev.) at 5 µM were 41 ± 3% (bienA9, N = 702), 12 ± 2% (bienA10, N = 808) and 1 ± 1% (bienA11, N = 813). At 10 µM, a similar trend was observed, with both bienA10 and bienA11 showing complete disruption of the entire vesicle population, while 19.9 ± 0.5% (N = 559) of the vesicles survived the 10 µM bienA9 treatment. These results are in excellent agreement with the corresponding antimicrobial activity observed in the single-cell experiments. In light of our previous studies with SLBs^23^, and the fact that the bienA peptides are hemolytic^23^, both our single-cell and single-GUV studies indicate a membranolytic mechanism of action for these peptides, with bienA11 being the most potent of the set.

In contrast, treatment with each of the bienK peptides led to considerable variability in vesicle survival within GUV populations, akin to the variability observed in the single-cell experiments. In the bienK9 and bienK10 experiments, at both 5 μM and 10 µM concentrations, over 40% of the GUVs survived the treatment with no dye leakage from the vesicle lumen (survival rates: bienK9: 41 ± 20% for 5 µM, N = 815; 47 ± 7% for 10 µM, N = 527; bienK10: 40 ± 7% for 5 µM, N = 952; 48 ± 10% for 10 µM, N = 821). For the bienK11 peptides, survival was even higher (survival rates: 70 ± 13% for 5 µM, N = 908; 78 ± 4% for 10 µM, N = 1088), although we note that the differences in vesicle survival shown in Fig. 6 amongst the bienK peptides are not statistically significant. This suggests that the bienK series is less membranolytic than the bienA peptides, which likely also explains the observation that the bienK peptides are not hemolytic^23^. This agrees with the mechanistic differences in membrane disruption by the two series observed in SLBs, where the bienA peptides formed transmembrane pores, while the bienK peptides caused fractal ruptures in the distal leaflet of the bilayer^23^. It is intriguing to speculate on the apparent membrane-to-membrane differences in the response to treatment by a bienK peptide, where we see a mix of GUV disruption and survival within a dosed population. We have confirmed that this is not due to any dependence of the bursting time on GUV radius, which may show slight variations within the GUV population (SI Fig. S5). Unlike cells, which display an inherent heterogeneity in gene, protein and lipid expression even within a clonal population, the GUVs being examined here all come from the same production batch within an experiment. These observations, coupled with the apparent affinity of bienK peptides for septating *E. coli* cells and the previous measurements on anionic SLBs^23^, suggest that the bienK peptides target bacterial membranes with a different mechanism to the bienA series, potentially exploiting weaknesses in the membranes of cells as they are undergoing division.

In addition to elucidating membrane-to-membrane differences in the membranolytic efficacy of a peptide within a vesicle population, we can also use our single-GUV resolution studies to reveal the *mode of action* of membrane-targeting antimicrobial peptides^45,46^. The mode of action is determined by studying the distribution of the time-points at which individual vesicles lose their fluorescence due to membrane disruption by the peptides, as previously described^45,46^. The heat-maps quantifying dye leakage from the vesicles (Figs. 5 and S3) demonstrate distinct time distributions of these single-GUV leakage events (LEs), which reveal the overall behaviour of the peptide-treated vesicle populations. In the cases of bienA11 and bienA10, the entire vesicle population exhibited LEs, meaning that all the individual GUVs’ membranes were disrupted (with 10 µM peptide). The LEs converged in a unimodal narrow distribution around a concentration-specific time-point. A similar trend was observed for bienA9, but in this case a subpopulation of vesicles survived at both treatment dosages. Based on the concentration dependent leakage behaviour, the distributions of the timings of the LEs and our recent studies correlating the mechanisms and modes of action of eleven membrane active peptides using the GUV assay, we suggest that the bienA series causes transmembrane disruptions that follow the two-state model described by Huang and others^46,47^. BienA11 and bienA10 thus subscribe to what is known as the unimodal “graded” mode of action while bienA9 follows the bimodal all-or-none mode^45^.

In contrast, in the bienK series, the LE data demonstrated similar membranolytic activity at both concentrations (Figs. 5 and S3). The LEs followed a stochastic time distribution. Further, we observed high GUV survival rates in response to all three bienK sequences. Thus the mode of action for the bienK peptides is associated with stochastic LEs where a large fraction of GUVs survive the treatment. This behaviour has previously been observed in a 2-helix substructure of the peptide epidermicin^46^. In a manner similar to the bienK peptides, the epidermicin substructure also caused interfacial defects at the outer leaflet of a lipid bilayer rather than transmembrane disruption^23,48^, strengthening the correlation between this membrane thinning molecular mechanism and the stochastic LE mode of action^46^.

## Conclusions

Our single-cell and single-GUV level studies of experimental peptides reveal the importance of quantifying variability in the antimicrobial activity of individual peptides within a bacterial and GUV population. Importantly, the use of our microfluidic platforms enables us to study hundreds of individual cells or GUVs within a single experiment, unlike earlier microscopy based methods that could typically study up to tens of individuals per experiment^49^. It is this capacity to probe the action of peptides on hundreds of individual cells/GUVs within the same experiment, under similar conditions, that allows us to comprehensively investigate the variability in the antimicrobial and/or membranolytic activity of these compounds within a cell or GUV population. Characterizing this variability in response to treatment with a specific peptide—at both the membrane and cellular level—will be key for the further translational development of these candidate antimicrobials. Our platforms may be used to guide iterations in peptide sequences to enhance antimicrobial activity, such as exploring the use of other amino acids (for example, arginine or tryptophan^50^) instead of alanine/lysine at the mutation position (Table 1). Further, the membrane and cellular-level variability we observed may translate into heterogeneous outcomes when such drugs are used to treat infections in vivo. Therefore, these peptide therapeutics will need to be carefully evaluated in pre-clinical models like those presented in this work to help design appropriate treatment strategies. More generally, our results add to the growing body of evidence that show some of the challenges associated with quantifying the activity of peptide-based therapeutics^16,21^. However, given the new strategy shown here and also complementary tools being developed elsewhere^21^, it is hoped that these difficulties can be quantified and addressed at an early stage of development, facilitating the efficient clinical translation of this promising category of therapeutics.

## Experimental section

### Materials and methods

#### Peptide synthesis

All peptides in the study were prepared as described elsewhere^23^. Briefly, the peptides were assembled on a Liberty microwave peptide synthesizer (CEM Corp.) using Fmoc/tBu solid-phase protocols and HBTU/DIPEA as coupling reagents. Rink amide 4-methylbenzhydrylamine resin was used for all the peptides. After cleavage and deprotection (95% TFA, 2.5% TIS, 2.5% water) the deprotection solutions were decanted using ether, and the peptides were spun down, freeze-dried and purified using a Thermo Scientific Dionex RP-HPLC system (Ultimate 3000). High performance liquid chromatography (HPLC) Vydac C18 analytical (5 mm) and semi-preparative (5 mm) columns were used. Analytical runs were performed with a 10–70% B gradient over 30 min at 1 mL/min and semi-preparative runs were optimised for each peptide at 4.7 mL/min. Aqueous CH_3_CN containing 0.1% TFA was used for buffer A (5%, v/v) and buffer B (95%, v/v). Detection was done at 230 and 214 nm. After post-synthesis treatments and purification using HPLC, the peptides were identified by matrix assisted laser desorption ionization time of flight (MALDI-ToF) mass spectrometry.

#### Cell culture

The *Escherichia coli* (BW25113) cultures were prepared in 150 mL of Lysogeny Broth (LB; 10 g/L tryptone, 5 g/L yeast extract and 10 g/L NaCl, Melford) with shaking at 200 rpm and incubation at 37 °C overnight (drug dosage during experiments was typically performed around 19–20 h after starting the culture). Prior to seeding in the microfluidic chip, the OD (optical density) of the culture was measured, the cells spun down at 3220*g* for 5 min and the supernatant filtered twice (0.22 µm, Millipore); this twice filtered supernatant is hereafter referred to as “spent LB”. All OD measurements were carried out at 600 nm. The cell pellet was resuspended in spent LB at an approximate OD of 75; this concentrated stationary phase culture was used when seeding the microfluidic chip with bacteria. Note, the OD of 75 is a theoretical value—we measure the OD of the original stationary phase culture, and calculate the volume of spent LB that the cell pellet (post centrifugation) is to be resuspended in to achieve this OD. In practical terms, one simply needs to concentrate the culture to optimise the efficiency of cells entering the wells of the mother-machine microfluidic device within a 30 min timeframe. We also note that unlike most traditional bulk experiments, we chose to study cells initially seeded in the microfluidic device in stationary phase, in line with previous studies using this platform^30–32,34^. Given that bacteria in natural environments can seldom sustain exponential growth, increasing efforts are being dedicated to understanding their behaviour in stationary phase^51^. Our methodology allows flexibility in this regard—the cells can be studied in stationary phase or can be grown on chip after seeding by supplying fresh nutrients, enabling comparisons of bacterial-drug interactions in a range of metabolic states^28^.

#### Microscopy for single-cell experiments

We used an Olympus IX73 epifluorescence microscope equipped with two Physik Instrumente piezo stages (M-545.USC and P-545.3C7) to control stage movement in XYZ. Images were acquired using an Olympus UPLSAPO 60× W objective (NA 1.2) and recorded on an Andor sCMOS (Zyla 4.2) camera with exposure times of 30 ms. We used the green LED of a pE-300 White LED light source (at 20% intensity) and a TRITC filter set for imaging the fluorescence of dead cells stained with propidium iodide (PI).

#### Polypeptide antibiotic preparation

Peptide stock solutions were always prepared fresh in milliQ water from the powders (stored at − 80 °C) before each experiment. The absorbance of the stock solution was measured in a NanoDrop 2000 spectrometer at 214 nm, and used to calculate the stock concentration based on the corresponding extinction coefficient. We typically measured the absorbance of the stock solution at least thrice and used the average value to calculate the concentrations. For the cell experiments, the stock was diluted to 10 μM in a solution of 90% minimal media and 10% fresh LB (by volume) for use in the experiments^52^, with a total volume of 400 µL. The minimal media consisted of 1× M9 salts, 2 mM MgSO_4_, 0.1 mM CaCl_2_ and 1 mg/L thiamine hydrochloride in milliQ water. For the control experiments, the dosage solution consisted of 30 µL of milliQ water (no peptide) in 370 µL of the minimal media-LB preparation. At each stage, the solutions were thoroughly vortexed to ensure complete mixing.

#### Microfluidics-microscopy assay for single-cell phenotyping

We recently published a detailed protocol of our microfluidics-microscopy assay, which is based upon the microfluidic “mother-machine” device pioneered by the Jun lab^29^; we have previously also used the device for quantifying survivor subpopulations in response to conventional small molecule antibiotics^30^. The device consists of a two-layer microfluidic chip with a main channel of width 100 μm and height 25 μm that is used for seeding the chip with bacteria, as well as nutrient, drug or dye dosing, along with thousands of smaller side channels, or “wells” (1.4 μm × 1.4 μm × 25 μm) which are used to physically confine the bacteria single-file for testing and long-term visualisation. The microfluidic chips are prepared from polydithmethylsiloxane (PDMS, Sylgard 184, Dowsil) by casting a 10:1 mixture of elastomer:curing agent (by weight) on an epoxy mold of the device (kindly provided by the Jun lab). This is cured for 2 h at 70 °C before being cut out, with inlet/outlet fluidic ports punched using a 0.75 mm biopsy punch (WellTech Rapid-Core). The chip is plasma bonded via a standard protocol (10 s plasma exposure, 30W, Zepto plasma oven, Diener Electric, Germany) to a type I glass cover slip following which the channels are passivated with bovine serum albumin (BSA, 50 mg/mL in milliQ water) at 37 °C for at least 30 min. The chip is then seeded with a concentrated solution (OD_600_ approximately 75) of stationary phase *E. coli* via a syringe and tubing (Portex, microfluidic fine bore polythene tubing 1.09 mm × 0.38 mm). The chip is left for 5–10 min at 37 °C to allow the bacteria to enter the small side channels (wells) of the device, following which it is connected to the microfluidic pump system; we use a 4-channel Fluigent MFCS pressure pump with an associated Fluigent Flow Unit, which enables us to set a desired flow rate during the course of the experiments via a feedback system with the pressure pump. Experimental solutions are housed in the Fluigent Fluiwell-4C holder, which enables the exchange of solutions via a simple exchange of vials (1.5 mL, Micrewtube) without any disturbance to the microfluidic device itself.

After plugging the tubing (Fluigent) from the flow unit into the inlet of the chip and connecting the outlet to a waste reservoir, the flow unit is first calibrated and then used to flow spent LB for 8 min at 300 μL/h through the chip, to flush out the concentrated bacterial solution from the main channel of the device. Following this, the flow is stopped and the spent LB vial exchanged for a vial containing 400 μL of the peptide solution (10 μM). We chose 10 μM as the peptide concentration since this is above the reported MIC values (in the range of 3–6 μM) for these peptides in *E. coli* (ATCC 15597) cells^23^. To initially effect solution exchange, whenever the solution vial is changed, we flow the new solution at 300 μL/h for 8 min through the chip, and then reduce the flow rate to 100 μL/h. The chip is thus treated with the antimicrobial for 3 h with bright-field images acquired at hourly intervals. Following 3 h of treatment, the antimicrobial solution is exchanged with fresh LB (8 min at 300 μL/h followed by a 100 μL/h flow rate), with images again acquired for 3 h at hourly intervals. After 3 h, the flow is reduced to 50 μL/h and left overnight (typically for around 14–15 h). Finally, the cells are treated with the stain Propidium Iodide (PI, 1:1000 dilution of the stock in LB) for 8 min at 300 μL/h followed by 15 min at 100 μL/h, after which the cells are imaged in both bright-field and fluorescence mode (TRITC filter set, green LED) to characterize cell death and survival at the end of the experiment. Dead cells and debris stain with PI and show fluorescence, whereas cells that are seen intact in bright-field and remain dark under fluorescence illumination are classified as being alive. These include both survivors that divide and fill the channel as well as non-dividers that we have characterized previously^30–32^.

All image analyses were performed manually using FIJI. Note that, as shown in Fig. 3, we often saw cells reaching the division point and then dying, which is why cells were only counted as having divided when it was clear that they were not still joined at the division site. This may in some cases lead to a slight underestimation of the division rate, but based on our experiments we noted that this was a more accurate representation of the results. This is also partly the reason we chose to analyse the images manually.

#### Microfluidics-microscopy assay for single-vesicle phenotyping

We have published detailed protocols for the design, fabrication and operation of the microfluidic device for immobilizing and testing thousands of individual GUVs, which can be found elsewhere^23,27^. A detailed protocol for the octanol-assisted liposome assembly (OLA) technique of producing GUVs on chip is also available^53^. Briefly, our platform facilitated three procedural steps integrated in a single device. First, the OLA^40^ technique was used to prepare GUVs. Next, the GUVs were trapped downstream in arrays of hydrodynamic posts. Finally, the immobilized GUVs were perfused with the desired dose of a peptide. All lipids described in the text were purchased from Avanti Polar Lipids. The GUVs were prepared in sucrose solution (200 mM) with glycerol (15% v/v) in PBS (pH 7.4). The vesicles were formed encapsulating the fluorescent membrane-impermeable dye 8-hydroxypyrene-1,3,6-trisulfonic acid from Thermo Fisher (HPTS, 50 μM). The microfluidic platform was operated by two positive pressure-driven pump modules (MFCS-4C, Fluigent), and a single neMESYS syringe pump module for fluid manipulation. The trapped GUVs were continuously dosed with buffer solution loaded with 5 μM HPTS (as a tracer) and the peptide of interest at 5 or 10 μM concentrations (the stock peptide solutions were prepared in milliQ water as described previously). Peptide arrival in the vesicle chamber, used to define the *t* = 0 time point, was monitored by tracking the HPTS tracer in the peptide perfusion solution. The data was later analysed using a custom Python code (available at https://github.com/mrcsfltchr/TrapAnalysis) that detected the GUVs and collated their fluorescence intensity traces with peptide arrival in the microfluidic chambers.

## Supplementary Information

Supplementary Information.

## Data Availability

All the data necessary for understanding the paper is available in the main text or supplementary information. Codes for the analysis of the GUV data are available at https://github.com/mrcsfltchr/TrapAnalysis.
