# Supplementary material for: An ultrasensitive microfluidic approach reveals correlations between the physico-chemical and biological activity of experimental peptide antibiotics
Source: Sci Rep. 2022 Mar 7;12:4005. doi: 10.1038/s41598-022-07973-z (PMC8901753; doi:10.1038/s41598-022-07973-z)
Supplement: Supplementary file 1 — Supplementary Information. [file 41598_2022_7973_MOESM1_ESM.pdf]

Supplementary Information

An ultrasensitive microfluidic approach reveals correlations between the physico-chemical and biological activity of experimental peptide antibiotics

Jehangir Cama[1,2,3]*, Kareem Al Nahas[3], Marcus Fletcher[3], Katharine Hammond[4,5], Maxim G. Ryadnov[4,6], Ulrich F. Keyser[3] and Stefano Pagliara[1,7]*

1. Living Systems Institute, University of Exeter, Stocker Road, Exeter EX4 4QD, UK
2. College of Engineering, Mathematics and Physical Sciences, University of Exeter, North Park Road, Exeter EX4 4QF, UK
3. Cavendish Laboratory, Department of Physics, University of Cambridge, JJ Thomson Avenue, Cambridge CB3 0HE, UK
4. National Physical Laboratory, Hampton Road, Teddington, Middlesex TW11 0LW, UK
5. London Centre for Nanotechnology, University College London, London WC1H 0AH, UK
6. Department of Physics, King's College London, Strand Lane, London WC2R 2LS, UK
7. College of Life and Environmental Sciences, University of Exeter, Stocker Road, Exeter EX4 4QD, UK

Correspondence: camajehangir@gmail.com, s.pagliara@exeter.ac.uk

Supplementary information includes:

- Tables S1-S4
- Figures S1-S5
- Supplementary references

**Treatment (10 μM)**	**Initial no. of cells (*t*=0)**	**No. of non-dividing survivors at the end of the experiments**
Bien A9	310	11
Bien A10	320	6
Bien A11	323	24
Bien K9	317	13
Bien K10	305	7
Bien K11	311	1

Table S1. Quantifying non-dividing survivors in response to treatment with the bien peptides. In response to our treatment (10 μM peptide), in addition to the results reported in the main manuscript, we observed a small number of *E. coli* cells that did not divide, but also did not stain with the dead-stain propidium iodide (PI) at the end of the experiments (Figure S1B). These cells are potentially survivors, akin to phenotypes we have reported previously[1–3].

**Statistical testing of differences in the Mean Survival Fractions: Cell experiments**	**p-value (2-sample t-test incorporating Welch's correction)**
Bien A9 vs Bien A10	0.0383
Bien A9 vs Bien A11	--- (mean and s.d. was 0 for A11)
Bien A10 vs Bien A11	--- (mean and s.d. was 0 for A11)
Bien K9 vs Bien K10	0.3671
Bien K9 vs Bien K11	0.80375
Bien K10 vs Bien K11	0.38824

Table S2. Statistical comparison of the mean survival fractions in the single-cell experiments (10 μM peptide dosage). Data analysed from 2 independent repeats, using a 2-sample t-test with Welch's correction to assess whether the means were significantly different at the 0.05 level (tested using Origin2019b software).

**Statistical testing of differences in the Mean Survival Fractions: GUV experiments (5 μM)**	**p-value (2-sample t-test incorporating Welch's correction)**
Bien A9 vs Bien A10	0.00986
Bien A9 vs Bien A11	0.01057
Bien A10 vs Bien A11	0.03013
Bien K9 vs Bien K10	0.93882
Bien K9 vs Bien K11	0.25652
Bien K10 vs Bien K11	0.12984

Table S3. Statistical comparison of the mean survival fractions in the single-vesicle experiments (5 μM peptide dosage). Data analysed from 2 independent repeats, using a 2-sample t-test with Welch's correction to assess whether the means were significantly different at the 0.05 level (tested using Origin2019b software).

**Statistical testing of differences in the Mean Survival Fractions: GUV experiments (10 μM)**	**p-value (2-sample t-test incorporating Welch's correction)**
Bien A9 vs Bien A10	--- (mean and s.d. was 0 for A10)
Bien A9 vs Bien A11	--- (mean and s.d. was 0 for A11)
Bien A10 vs Bien A11	N/A (mean and s.d. were 0 for both)
Bien K9 vs Bien K10	0.89778
Bien K9 vs Bien K11	0.05308
Bien K10 vs Bien K11	0.1148

Table S4. Statistical comparison of the mean survival fractions in the single-vesicle experiments (10 μM peptide dosage). Data analysed from 2 independent repeats, using a 2-sample t-test with Welch's correction to assess whether the means were significantly different at the 0.05 level (tested using Origin2019b software).

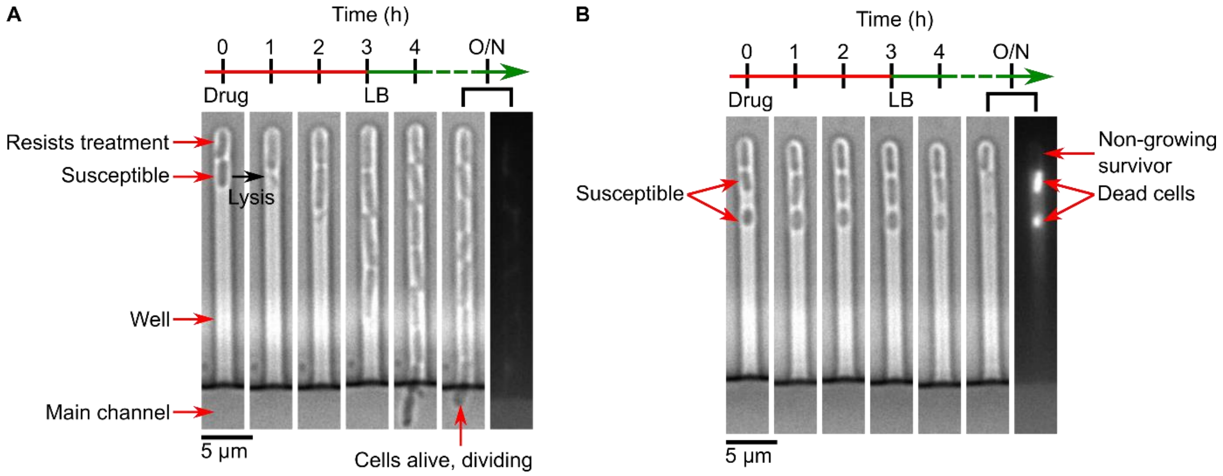

Figure S1. Representative images showing the different cellular phenotypes observed in response to peptide treatment. In (A), the images depict two *E. coli* cells trapped in a microfluidic well (at $t = 0$), subjected to 10 µM of the bienA9 peptide for 3 h, followed by fresh nutrient delivery (LB). The panel shows bright-field microscopy images taken at hourly intervals during peptide treatment, followed by bright-field images after 1 h and overnight (O/N) growth in fresh LB. The last panel shows the cells (fluorescence imaging) after treatment with the dead stain propidium iodide (PI). The initial two (clonal) cells are from the same culture and exposed to identical treatments. As can be seen, the cell at the bottom lysed upon exposure to the peptide. In stark contrast, its neighbour resisted the peptide, growing and dividing through the treatment. The daughter cells continued dividing thereafter in fresh LB media (cells post overnight growth were alive and did not stain with PI). In (B), we track the response of 3 individual, clonal *E. coli* cells to the bienA10 peptide (10 µM). None of the cells divided either during treatment or after fresh LB media was flushed through the device. However, only 2 of the cells died and stained with PI. These cells also disintegrated after the overnight LB treatment. In contrast, the topmost cell in the images did not disintegrate, nor did it stain with PI. Yet it did not divide, akin to the so-called “viable but non culturable” (VBNC) phenotype that we have characterized previously[1–3]. We label such cells “non-dividing survivors” for the purposes of this paper.

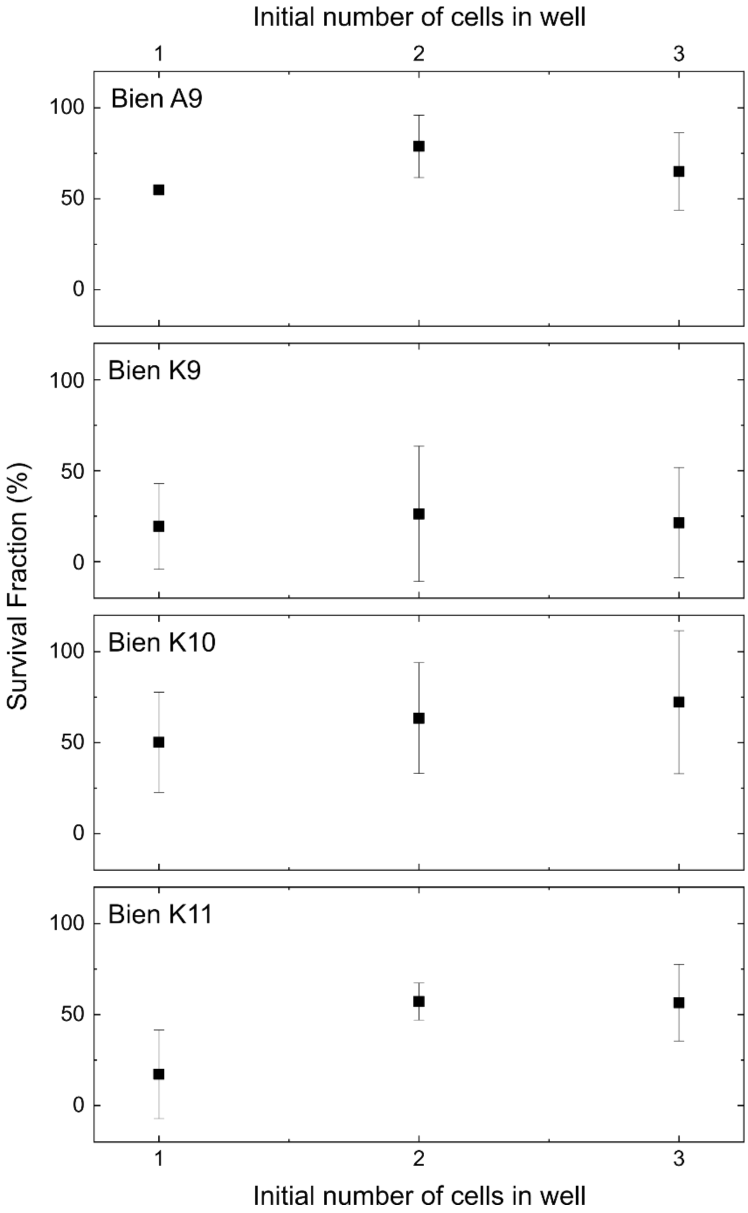

Figure S2. Comparing the effect of initial cell numbers on survival fractions (peptide dosage 10 μM). As mentioned in the main text, apart from the bien A10 and bien A11 peptides that showed near uniform growth inhibition and killing of the cell populations, for the remaining peptides we analysed wells that hosted between 1-3 *E. coli* cells at the start of the experiment. In this figure, we explore whether the initial cell number influences the survival fraction. For bien A9, bien K9 and bien K10, the survival fractions remain similar as the initial number of cells in the wells increased from 1 to 3. For bien K11 although at first glance there appears to be some protective effect of having more than 1 cell in the well, the difference is not statistically significant at the 0.05 level (p-value 0.22523; 2-sample t-test with Welch's correction, comparing the 1-cell and 2-cell data; for comparing the 1-cell and 3-cell data, the p-value is 0.2286). Data points report the means and s.d. from 2 independent repeats for each peptide. Thus based on the data we have gathered, there appears to be no relevant inoculum effect on the survival fraction when the wells were initially loaded with 1,2 or 3 cells.

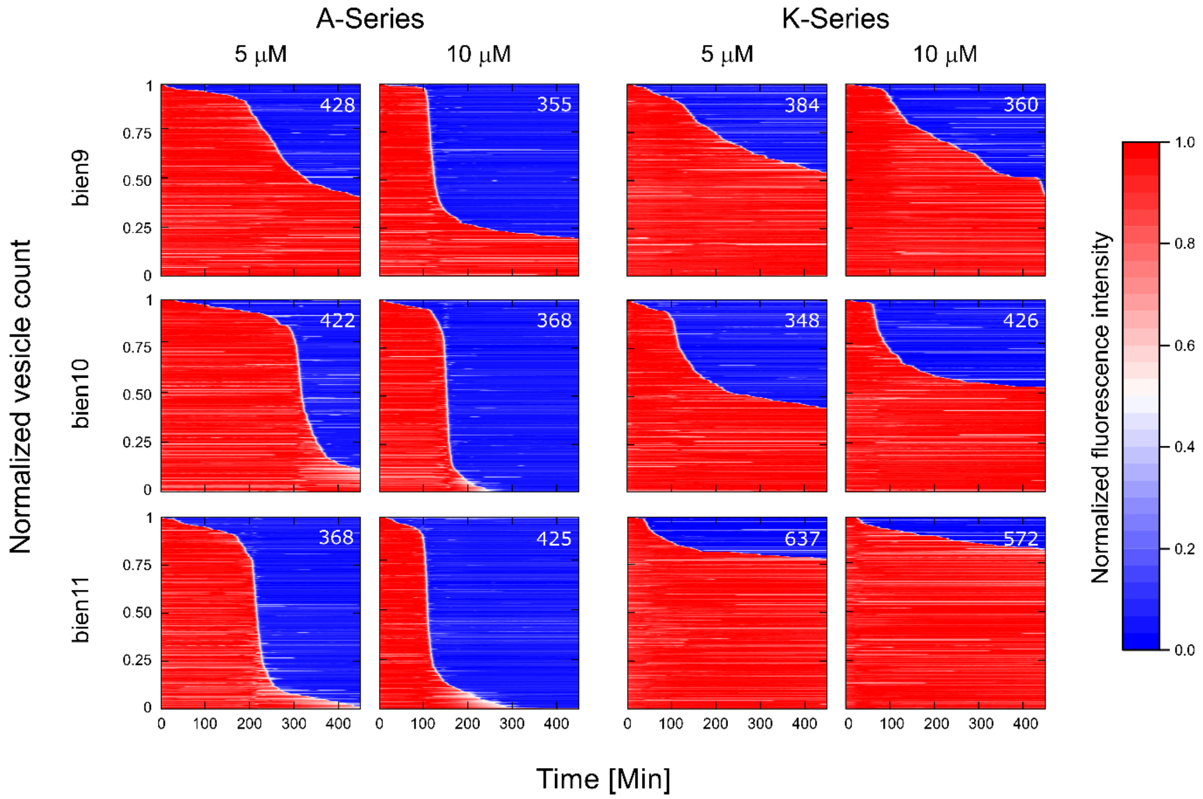

Figure S3. A summary of the membranolytic activity of the bien peptides on populations of bacterial membrane-mimicking lipid vesicles. For completeness, includes data that has been reprinted (adapted) with permission from K. Hammond, F. Cipcigan, K. Al Nahas *et al.*, "Switching Cytolytic Nanopores into Antimicrobial Fractal Ruptures by a Single Side Chain Mutation", *ACS Nano*, 15 (6), 9679-9689 (2021). Copyright 2021 American Chemical Society. Trapped vesicles were continuously treated with the peptides at 5 µM and 10 µM concentrations and their morphology was observed overnight. Each horizontal line depicts the locally normalized intensity of the fluorescent dye HPTS encapsulated in a single trapped vesicle (with global background subtraction) over time. The vesicle's membrane is considered intact at high fluorescence intensity (red) and compromised at low fluorescent signal (blue). The intensity traces were ordered by the critical viability time point, which is defined as the point when the fluorescence intensity of a vesicle decreases below 50% of its initial intensity. The total number of analysed vesicles is reported in white in the top right corner of every plot. The results show that the bienA series of peptides is membranolytic, with potency increasing as one progresses from bienA9 to bienA11 and with an increase in the respective drug concentrations. However, the bienK series is not obviously membranolytic – there appears to be a weakening of the membranes in relation to controls, but there is no obvious concentration dependence, and the trend appears to be different compared to the bienA series, with bienK11 being the least potent in this dataset.

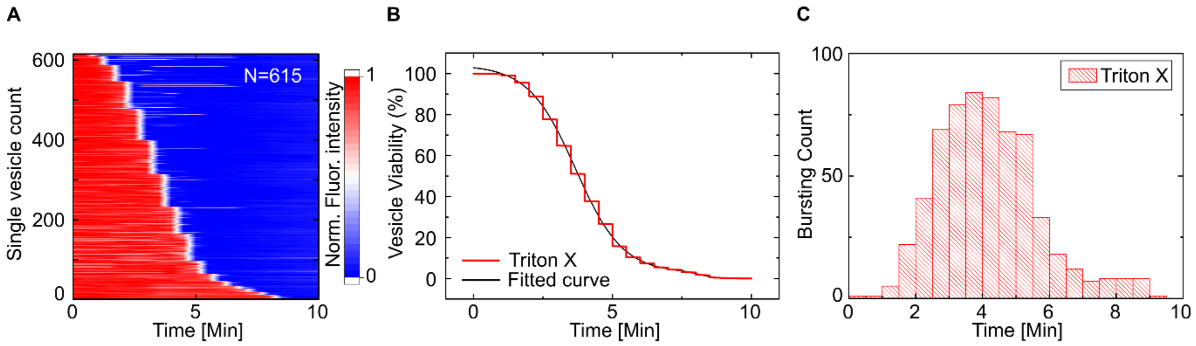

Figure S4. Positive control experiment using the GUV platform[4], analysing the effect of the detergent Triton X (0.06% (w/v)) on our model bacterial membrane-mimicking lipid vesicles. (A) Heat-map showing the strong membranolytic activity of Triton X, which lysed all the vesicles (N = 615) within 10 minutes. (B) Tracking vesicle viability after Triton X exposure confirms rapid GUV lysis. The stair shaped dataset was fitted with a Double Boltzmann sigmoidal fit. (C) Histogram depicting the Leakage Event time distribution of the trapped vesicles. Figure adapted from KAN's first year PhD (CPGS, University of Cambridge) progress report.

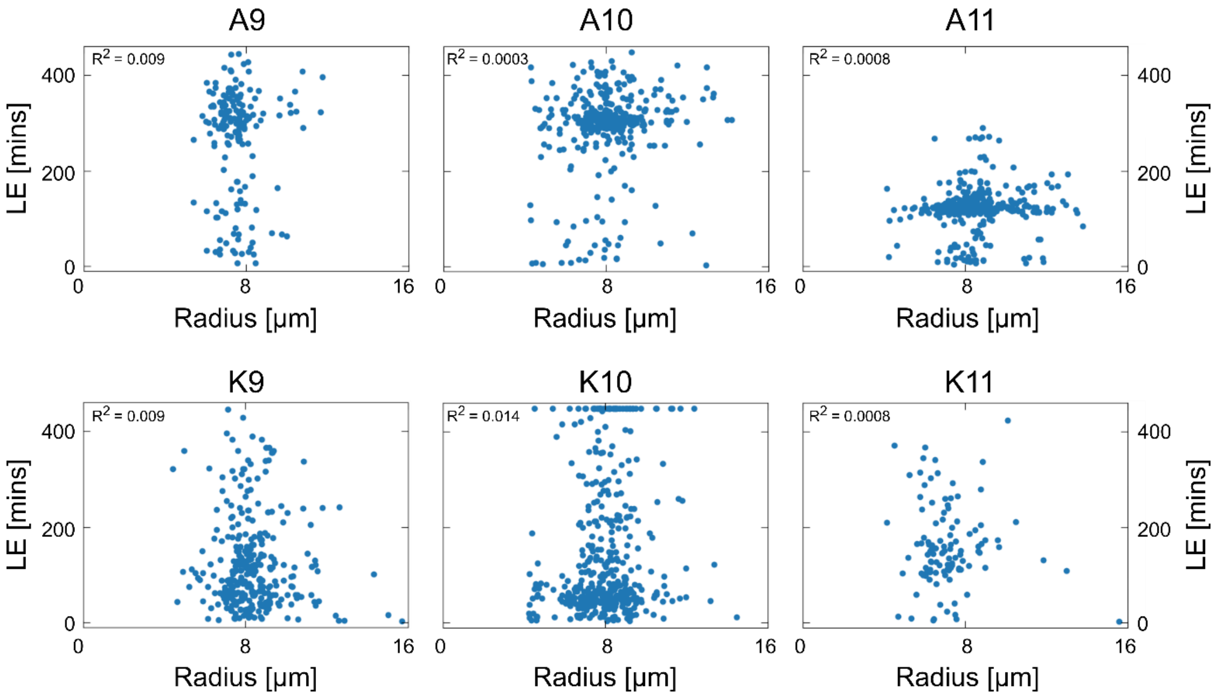

Figure S5. No correlation observed between GUV radius and time of bursting/leakage. The plots represent data from experiments performed using the 6 bien peptides in our study at 5 µM concentrations on our model bacterial membrane-mimicking vesicles. Each data point refers to a vesicle that exhibited a leakage event (LE). For clarity, vesicles that survived through the entire experimental timescale are not shown. Across all the peptides, we observed no correlation between the timing of the leakage event and GUV radius. R^2 values showing the lack of any correlation are provided inset in each plot.

Supplementary references:

(1) Goode, O.; Smith, A.; Zarkan, A.; Cama, J.; Invergo, B. M.; Belgami, D.; Caño-Muñiz, S.; Metz, J.; O'Neill, P.; Jeffries, A.; Norville, I. H.; David, J.; Summers, D.; Pagliara, S. Persister Escherichia Coli Cells Have a Lower Intracellular PH than Susceptible Cells but Maintain Their pH in Response to Antibiotic Treatment. *MBio* **2021**, *12* (4), e00909-21.

(2) Goode, O.; Smith, A.; Łapińska, U.; Bamford, R.; Kahveci, Z.; Glover, G.; Attrill, E.; Carr, A.; Metz, J.; Pagliara, S. Heterologous Protein Expression Favors the Formation of Protein Aggregates in Persister and Viable but Nonculturable Bacteria´. *ACS Infect. Dis.* **2021**, *7*, 1848–1858.

(3) Bamford, R. A.; Smith, A.; Metz, J.; Glover, G.; Titball, R. W.; Pagliara, S. Investigating the Physiology of Viable but Non-Culturable Bacteria by Microfluidics and Time-Lapse Microscopy. *BMC Biol.* **2017**, *15*, 121.

(4) Al Nahas, K.; Cama, J.; Schaich, M.; Hammond, K.; Deshpande, S.; Dekker, C.; Ryadnov, M. G.; Keyser, U. F. A Microfluidic Platform for the Characterisation of Membrane Active Antimicrobials. *Lab Chip* **2019**, *19*, 837–844.
